# Blood urea nitrogen-to-albumin ratio predicts mortality in acute graft-versus- host disease after allogeneic stem cell transplantation

**DOI:** 10.3389/fimmu.2026.1796065

**Published:** 2026-07-16

**Authors:** Gangping Li, Di Zhang, Minghui Li, Yongqi Wang, Fangfang Yuan, Jian Zhou, Yuewen Fu

**Affiliations:** 1 Department of Hematology, The Affiliated Cancer Hospital of Zhengzhou University & Henan Cancer Hospital, Zhengzhou, China; 2 Department of Medical Records Management, The Affiliated Cancer Hospital of Zhengzhou University & Henan Cancer Hospital, Zhengzhou, China

**Keywords:** acute graft-versus-host disease, albumin, allogeneic hematopoietic stem cell transplantation, blood urea nitrogen, prognostic

## Abstract

**Background:**

Evidence regarding the association between the blood urea nitrogen to albumin ratio (BAR) and clinical outcomes in acute graft-versus-host disease (aGVHD) following allogeneic hematopoietic stem cell transplantation (allo-HSCT) remains limited.

**Objective:**

This study aimed to evaluate the prognostic significance of BAR levels in patients developing aGVHD following allo-HSCT.

**Methods:**

We performed a retrospective cohort analysis of allo-HSCT recipients at Henan Cancer Hospital (January 2019-December 2024). Eligible participants were diagnosed with aGVHD during post-transplantation follow-up. Demographic characteristics, BAR measurements, and clinical outcomes were extracted from electronic medical records. Primary endpoints comprised all-cause mortality (ACM) and non-relapse mortality (NRM). Multivariable Cox proportional hazards models with confounder adjustment and subgroup analyses were employed to assess mortality associations.

**Results:**

Among 109 included patients (mean age 29.4 ± 15.3 years), 51 presented with grade I-II aGVHD and 58 with grade III-IV aGVHD. During the 30-month follow-up, 69 deaths, 3 relapse/progression events, and 37 survivors were documented. Elevated BAR (continuous) independently predicted increased all-cause mortality (ACM) (HR=5.92, 95% CI 1.66–9.16; p=0.006) and non-relapse mortality (NRM) (HR=5.26, 95% CI 1.48–8.71; p=0.010). Tertile analysis (T3 vs. T1) showed higher ACM (HR=2.19, 95% CI 1.05–4.57; p=0.037) and NRM (HR=2.07, 95% CI 1.01–4.22; p=0.046), with significant dose-response trends (p<0.05). Kaplan-Meier analysis confirmed inferior survival in high-BAR groups (OS p=0.0091; RFS p=0.015). Subgroup analyses demonstrated consistent effects across age, sex, conditioning regimens, graft types, and ABO compatibility (interaction *p*>0.05). Sensitivity analyses confirmed robustness of these associations.

**Conclusion:**

Elevated BAR levels independently predict increased mortality in allo-HSCT recipients with aGVHD, suggesting its utility as a pragmatic prognostic biomarker requiring prospective validation.

## Introduction

Allogeneic hematopoietic stem cell transplantation (allo-HSCT) remains a potentially curative therapy for various hematological malignancies and bone marrow failure syndromes (1–3). Despite advancements in conditioning regimens and supportive care, acute graft-versus-host disease (aGVHD) remains a formidable barrier to successful outcomes, affecting 30-50% of recipients (4–7).This immune-mediated complication, driven by donor T-cell reactivity against host tissues, significantly contributes to non-relapse mortality (NRM) and long-term morbidity (8). Consequently, the early identification of patients at high risk for severe aGVHD and its sequelae is paramount for implementing timely, targeted interventions and improving survival (9).

Significant research efforts have focused on identifying reliable biomarkers for aGVHD risk stratification and prognostication. Kidney damage demonstrates significant prognostic value for survival in allo-HSCT patients requiring ICU admission during the peri-transplant period (10). Hypoalbuminemia prior to allogeneic transplantation for acute myeloid leukemia and myelodysplastic syndromes showed a stronger association with inferior survival (11). Biomarkers reflecting tissue damage (e.g., Regenerating islet-derived protein 3-alpha [REG3α]) (6), inflammatory cytokines (e.g., Interleukin-6 [IL-6]) (6, 12), and biomarkers associated with endothelial dysfunction or gastrointestinal integrity (e.g., Suppression of Tumorigenicity 2 [ST2]) (6) have demonstrated prognostic utility. The Mount Sinai Acute GVHD International Consortium (MAGIC) algorithm, incorporating ST2 and REG3α, exemplifies progress in predictive modeling (6). However, the widespread clinical adoption of these novel biomarkers is often hindered by cost, assay complexity, and limited availability outside specialized centers (6, 13). There remains a compelling need for readily accessible, cost-effective prognostic indicators derived from routine clinical parameters.

The blood urea nitrogen to serum albumin ratio (BAR), an easily calculable metric from standard laboratory tests, has recently garnered attention as a potential prognostic indicator in critical illnesses like sepsis and heart failure (14, 15). This ratio potentially integrates two pathophysiological axes relevant to aGVHD: renal impairment (reflected by elevated BUN) and systemic inflammation/catabolic state (reflected by hypoalbuminemia) (14, 16). Renal dysfunction, often linked to systemic inflammation and endothelial activation, is increasingly recognized as a feature of severe aGVHD impacting outcomes (17). Concurrently, hypoalbuminemia is a well-established marker of malnutrition, inflammation, and disease severity in hospitalized patients, including those post-HSCT (18). Given the intricate interplay between inflammation, endothelial injury, and metabolic derangements in aGVHD pathogenesis (18), the BAR may serve as a composite surrogate reflecting this pathophysiological nexus. Despite its potential, the prognostic significance of the BAR specifically within the context of aGVHD following allo-HSCT remains largely unexplored. This retrospective cohort study aims to rigorously evaluate the association between the BAR at aGVHD onset and key clinical outcomes, thereby assessing its utility as a novel, accessible prognostic tool in this setting.

## Methods

### Patients and study design

**Figure 1:**
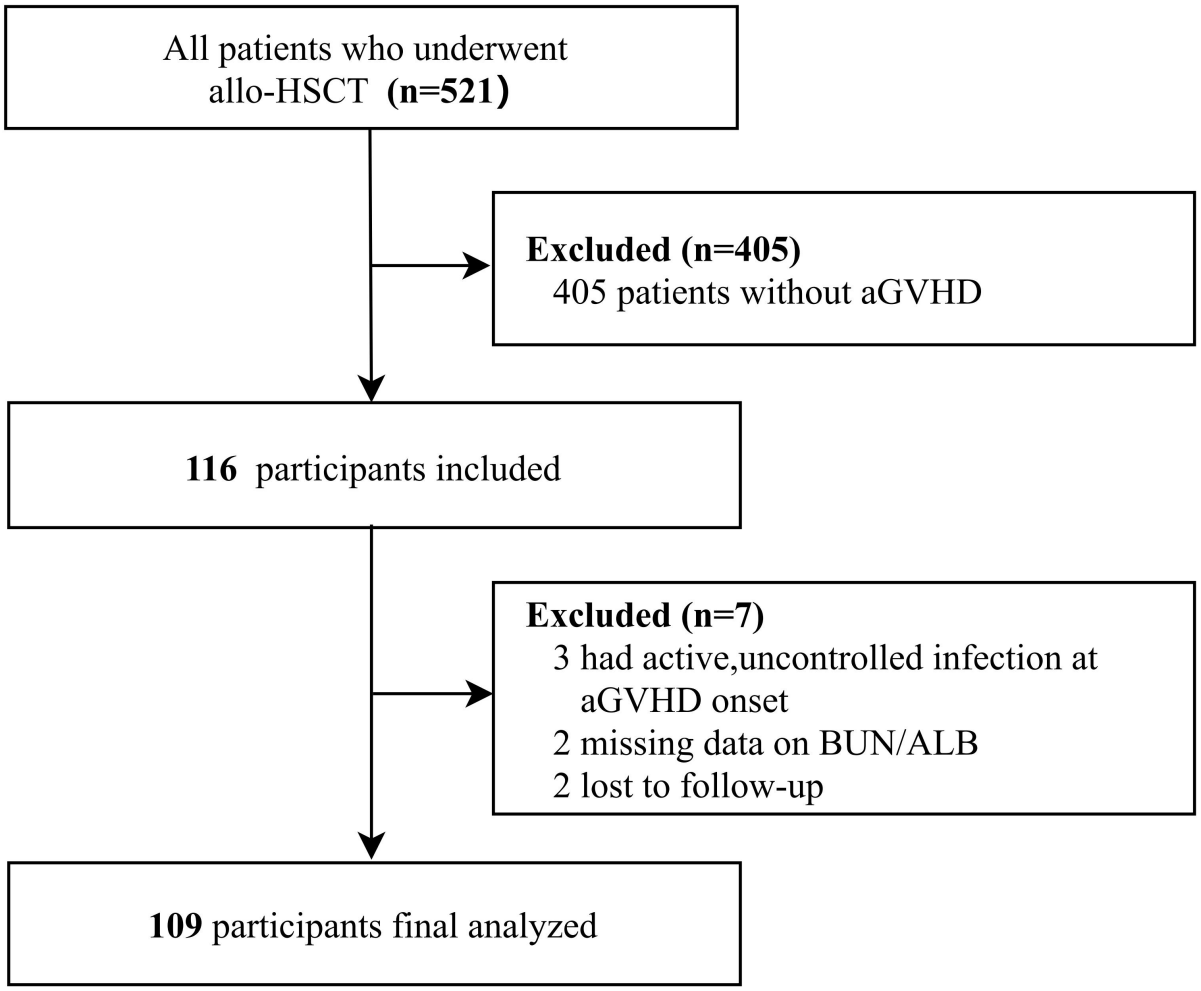
Flowchart of participant enrollment and procedures.

This retrospective cohort study included 521 consecutive patients undergoing allogeneic hematopoietic stem cell transplantation (allo-HSCT) at Henan Cancer Hospital between January 2019 and December 2024. Within this cohort, 116 patients developed acute graft-versus-host disease (aGVHD). Following exclusion of seven patients due to incomplete data or loss to follow-up, 109 patients with aGVHD were included in the final analysis cohort (**Figure 1**). The analysis adhered to a predefined plan focused on the association between BAR and mortality in aGVHD. All endpoints and covariates were specified prior to data extraction. This study was approved by the Institutional Review Board of the Affiliated Cancer Hospital of Zhengzhou University & Henan Cancer Hospital (Approval Number: 2022-060-001). All procedures complied with relevant guidelines and regulations and the Declaration of Helsinki.

### Conditioning regimens and GVHD prophylaxis

Patients receiving hematopoietic stem cells from unrelated or haploidentical donors underwent conditioning incorporating anti-human thymocyte immunoglobulin (ATG), whereas sibling donor transplants proceeded without ATG. The standardized protocol encompassed peripheral blood or bone marrow stem cell procurement, an ATG-based preparative regimen (where applicable), and graft-versus-host disease (GVHD) prophylaxis consisting of methotrexate, mycophenol mofetil (MMF), and cyclosporine A (CsA). Intravenous CsA (2.5–3 mg/kg/day) commenced on day -5, continuing until gastrointestinal recovery permitted transition to oral administration. Whole-blood CsA concentrations were assessed weekly via fluorescence polarization immunoassay (Cayman Chemical, Ann Arbor, MI), targeting levels of 150–250 ng/mL. CsA tapering began after day +180 in the absence of GVHD, except for severe aplastic anemia (SAA) cases where it was deferred for one year. MMF dosing followed a defined schedule: 15 mg/kg every 12 hours from day -5 to day +30, reduced by 50% thereafter, and discontinued by day +60. Methotrexate was delivered intravenously at 15 mg/m² on day +1 and 10 mg/m² on days +3,6, and +11. First-line therapy for acute GVHD involved corticosteroids (methylprednisolone 1 mg/kg/day). Patients exhibiting an inadequate response to steroids received salvage therapy with the anti-CD25 monoclonal antibody and ruxolitinib.

### Definitions

aGVHD severity classification followed consensus-modified Glucksberg criteria (14). All-cause mortality(ACM): The composite endpoint encompassing death from any cause, including disease progression, treatment complications, infections, or unrelated events (19). This metric captures overall survival (OS) without distinguishing specific etiologies. Non-Relapse Mortality (NRM): Death attributable to causes other than primary disease relapse or progression, typically encompassing treatment-related toxicities (e.g., graft-versus-host disease, organ failure, infections) or non-malignant comorbidities. NRM excludes mortality directly linked to underlying malignancy recurrence (20).

### BAR

The blood urea nitrogen to serum albumin ratio (BAR) was calculated as serum blood urea nitrogen (BUN)(mmol/L) divided by serum albumin (ALB) (g/L) measured within 48 hours of aGVHD diagnosis. Serum blood urea nitrogen (BUN) concentrations were determined using the Beckman Coulter AU5800 clinical chemistry analyzer (Beckman Coulter, Brea, CA, USA) employing the standardized urease-glutamate dehydrogenase (GLDH) coupled enzymatic assay. Serum albumin was quantified using the Beckman Coulter AU5800 analyzer (Beckman Coulter, USA) via bromocresol green methodology.

### Supportive treatment

All transplant recipients received comprehensive prophylaxis against infectious complications and treatment-related toxicities. The antiviral regimen included ganciclovir, while antifungal coverage was provided by either posaconazole or voriconazole. *Pneumocystis jirovecii* prophylaxis consisted of sulfamethoxazole. Additional protective measures comprised phenytoin for neuroprotection during busulfan administration, mesylate for hemorrhagic cystitis prevention, and ursodeoxycholic acid for hepatic veno-occlusive disease risk reduction. Standard supportive care incorporated hydration protocols, urine alkalinization, irradiated blood product transfusions, and other individualized interventions. Hematopoietic growth factors (subcutaneous recombinant human G-CSF and thrombopoietin) were administered from day +4 until hematopoietic recovery. Cytomegalovirus (CMV) and Epstein-Barr virus (EBV) viremia were quantified twice weekly during the inpatient period. Preemptive anti-CMV therapy (ganciclovir and foscarnet) was triggered when CMV DNAemia exceeded 1000 copies/mL. For EBV reactivation (≥5000 copies/mL on consecutive measurements), rituximab (100 mg) was administered.

In this study, sepsis was defined per Sepsis-3 criteria (infection plus acute SOFA score increase ≥2) (21). Severity was evaluated using SOFA, APACHE II. ICU management followed sepsis bundles, including early antibiotics, hemodynamic support, and organ-specific care. Specific clinical details-such as antibiotic selection, fluid strategies, and ventilation parameters-were not specified and were to be individualized per guidelines and patient factors. For aGVHD management, first-line therapy refers to methylprednisolone at 1-2 mg/kg/d. Steroid refractoriness is defined as disease progression after 3 days or incomplete response by day 7 (22). Second-line options include extracorporeal photopheresis (45% of cases), ruxolitinib (30%), or mesenchymal stromal cells (25%).

### Statistical analysis

The normality of distribution for all variables was assessed through three complementary approaches: histogram visualization, quantile-quantile plots, and the Kolmogorov-Smirnov goodness-of-fit test. Continuous variables demonstrating normal distribution were summarized using arithmetic mean values with standard deviation (mean ± SD), while non-normally distributed continuous variables were characterized by their median values accompanied by interquartile ranges (median [IQR]). Discrete variables were quantified as percentage frequencies within each category. Intergroup analyses were performed according to variable characteristics:

Categorical data comparisons employed either Pearson’s chi-square test or Fisher’s exact probability test, as appropriate for expected cell frequencies. Normally distributed continuous measures were evaluated using parametric one-way analysis of variance. Nonparametric Kruskal-Wallis rank-sum tests were applied to analyze skewed continuous variables across BAR classifications.

The association between blood urea nitrogen-to-albumin ratio (BUN/ALB) and mortality was quantified via Cox proportional hazards regression, reporting hazard ratios (HR) with 95% confidence intervals (CI). All models adjusted for clinically relevant covariates. Proportional hazards assumptions were validated through log-log survival plots and time-dependent interaction terms. Mortality outcomes were visualized using Kaplan-Meier curves stratified by BAR tertiles (T1-T3), with between-group differences assessed via log-rank testing. Patients were categorized into three risk strata based on cohort distribution:T1 (low risk): BAR 0.03–013; T2 (intermediate risk): BAR 0.13-027; T3 (high risk): BAR 0.2-1.32. This tertile-based stratification approach (14) enables clinically actionable risk discrimination. Follow-up discontinuations were handled by censoring at the last confirmed contact. Covariate selection followed *a priori* criteria incorporating clinical relevance, prior evidence univariate significance (p<0.05), and ≥10% impact on effect estimates.

Three sequential Cox regression models were developed to assess the BUN/ALB-mortality association: Model 1: Adjusted for demographic factors (age, sex); Model 2: Incorporated Model 1 covariates plus transplantation parameters (indication, stem cell source donor type, conditioning regimen, HSCT-to-diagnosis interval, ABO compatibility, MNC/CD34+ counts); Model 3: Expanded Model 2 with clinical metrics (engraftment kinetics, CMV/EBV viremia, hematologic parameters liver/renal function, infectious complications aGVHD severity). Exposure-response trends were evaluated by assigning numerical scores (median tertile values) to BAR categories in multivariable models. Prespecified subgroup explorations were stratified by relevant effect modifiers. Complete-case methodology was applied to the primary analysis, excluding records with missing data. Comprehensive sensitivity analyses examined result stability across alternative inference frameworks. All derived effect estimates and corresponding p-values were systematically documented and compared. Statistical analyses were performed using R4.2.2 (http://www.R-project.org, The R Foundation). Statistical significance was defined as two-tailed p < 0.05.

## Results

### Baseline characteristics

This study included 109 eligible patients with a mean age of 29.4 ± 15.3 years. During a follow-up period of 30 months, 69 deaths, 3 cases of disease relapse/progression, and 37 patients remained alive. Among the participants, 51 had Grade I-II aGVHD, and 58 had Grade III-IV aGVHD. **Table 1** presents the general characteristics of the participants stratified by BAR levels. Significant differences were observed across the three groups in terms of indication for HSCT, type of transplantation, pulmonary infection, febrile neutropenia, platelets, total bilirubin, creatinine, status, time from stem cell infusion to death from any cause or relapse/progression or death and aGVHD grade (all p < 0.05).

**Table 1:** The baseline characteristics and demographic characteristics of the study population by categories of BAR.

Characteristic	BAR				
	Total (n = 109)	1 (n = 36)	2 (n = 36)	3 (n = 37)	*P*-value
Age, Mean ± SD	29.4 ± 15.3	28.8 ± 13.8	25.7 ± 15.6	33.7 ± 15.6	0.076
Sex, n (%)					0.164
Male	68 (62.4)	18 (50)	24 (66.7)	26 (70.3)	
Female	41 (37.6)	18 (50)	12 (33.3)	11 (29.7)	
Indication for HSCT, n(%)					0.008
AML	29 (26.6)	17 (47.2)	7 (19.4)	5 (13.5)	
ALL	34 (31.2)	8 (22.2)	10 (27.8)	16 (43.2)	
MDS	16 (14.7)	4 (11.1)	8 (22.2)	4 (10.8)	
SAA	20 (18.3)	2 (5.6)	8 (22.2)	10 (27)	
Others	10 (9.2)	5 (13.9)	3 (8.3)	2 (5.4)	
Stem cell sources, *n* (%)					0.317
Bone marrow	103 (94.5)	36 (100)	33 (91.7)	34 (91.9)	
Peripheral blood	5 (4.6)	0 (0)	2 (5.6)	3 (8.1)	
Bone marrow + Peripheral blood	1 (0.9)	0 (0)	1 (2.8)	0 (0)	
Type of transplantation, *n* (%)					0.022
Unrelated match	50 (45.9)	9 (25)	22 (61.1)	19 (51.4)	
HLA match related	35 (32.1)	14 (38.9)	9 (25)	12 (32.4)	
Haplo-identical related	24 (22.0)	13 (36.1)	5 (13.9)	6 (16.2)	
Conditioning regimen, *n* (%)					0.361
MAC	56 (51.4)	15 (41.7)	20 (55.6)	21 (56.8)	
RIC	53 (48.6)	21 (58.3)	16 (44.4)	16 (43.2)	
ABO match, *n* (%)					0.131
Matched	42 (38.5)	18 (50)	14 (38.9)	10 (27)	
Unmatched	67 (61.5)	18 (50)	22 (61.1)	27 (73)	
Disease status before HSCT, *n* (%) ^a^					0.073
CR	70 (64.2)	29 (80.6)	22 (61.1)	19 (51.4)	
PR or NR	18 (16.5)	5 (13.9)	6 (16.7)	7 (18.9)	
MRD before HSCT, *n* (%) ^a,b^	21 (19.3)	2 (5.6)	8 (22.2)	11 (29.7)	
Positive					0.141
Negative	68 (62.4)	27 (75)	22 (61.1)	19 (51.4)	
ABO match, *n* (%)	21 (19.3)	7 (19.4)	6 (16.7)	8 (21.6)	
Matched	20 (18.3)	2 (5.6)	8 (22.2)	10 (27)	
MNC count, ×10^8^/Kg, Mean ± SD	13.4 ± 6.5	12.1 ± 6.6	14.7 ± 7.0	13.4 ± 5.8	0.239
CD34^+^ cells count, ×10^6^/Kg, Mean ± SD	6.8 ± 4.2	6.8 ± 5.8	6.5 ± 2.8	7.0 ± 3.6	0.896
Granulocyte implantation time, days, Mean ± SD	12.5 ± 2.3	12.7 ± 2.0	12.5 ± 2.0	12.4 ± 2.8	0.884
Days from transplantation to diagnosis	49.0 (20.0, 100.0)	42.0 (18.8, 87.8)	62.5 (37.2, 126.8)	54.0 (16.0, 129.0)	0.252
CMV viremia, n (%)	24 (22.0)	5 (13.9)	10 (27.8)	9 (24.3)	0.334
EBV viremia, n (%)	18 (16.5)	6 (16.7)	4 (11.1)	8 (21.6)	0.481
Pulmonary infection, n (%)	81 (74.3)	20 (55.6)	30 (83.3)	31 (83.8)	0.007
Intestinal infection, n (%)	58 (53.2)	15 (41.7)	22 (61.1)	21 (56.8)	0.221
Febrile neutropenia, n (%)	31 (28.4)	8 (22.2)	17 (47.2)	6 (16.2)	0.008
White blood cells, ×10^9^/L, Median (IQR)	2.5 (1.1, 4.4)	2.5 (1.5, 3.5)	2.3 (1.1, 4.5)	2.0 (0.7, 5.4)	0.95
Hemoglobin, g/L, Mean ± SD	87.2 ± 21.0	92.6 ± 17.6	84.2 ± 21.9	84.8 ± 22.6	0.17
Platelets, ×10^9^/L, Median (IQR)	37.0 (22.0, 72.0)	54.5 (30.8, 85.8)	33.5 (19.8, 69.0)	28.0 (15.0, 56.0)	0.011
Total bilirubin, (umol/L), Median (IQR)	17.8 (11.2, 33.4)	12.2 (9.6, 21.9)	16.9 (12.6, 36.9)	30.6 (15.9, 43.8)	< 0.001
Creatinine (mmol/L), Median (IQR)	51.0 (35.0, 84.0)	36.0 (28.0, 49.0)	45.0 (36.5, 59.2)	90.0 (70.0, 125.0)	< 0.001
Status, n (%)					0.037
Alive	37 (33.9)	18 (50)	10 (27.8)	9 (24.3)	
All-cause mortality	69 (63.3)	16 (44.4)	25 (69.4)	28 (75.7)	
Disease relapse/progression	3 (2.8)	2 (5.6)	1 (2.8)	0 (0)	
Non-relapse mortality	66(60.6)	14 (38.9)	24 (66.7)	28(75.7)	
Time, months, Median (IQR)					
from stem cell infusion to death from any cause	6.0 (3.0, 18.0)	11.5 (4.8, 25.2)	6.5 (4.5, 16.5)	5.0 (3.0, 7.0)	0.04
from stem cell infusion to relapse/progression or death	6.0 (3.0, 16.0)	11.0 (4.8, 23.0)	6.5 (4.5, 15.2)	5.0 (3.0, 7.0)	0.045
aGVHD, n (%)					0.01
Grade I-II	51 (46.8)	24 (66.7)	15 (41.7)	12 (32.4)	
Grade III-IV	58 (53.2)	12 (33.3)	21 (58.3)	25 (67.6)	

^a^ Indicates that patients with n=20 SAA were excluded from the MRD and disease status statistics before HSCT.

^b^ MRD was measured by flow cytometry before HSCT and MRD levels≥10^−4^ was defined as positive.

BAR, BUN/ALB; T1, BAR (0.03-0.13); T2, BAR (0.13-0.27); T3, BAR(0.27-1.32); SD, Standard Deviation; IQR, Interquartile Range; AML, acute myelocytic leukemia; ALL, acute lymphocytic leukemia; MDS, myelodysplastic syndrome; SAA, severe aplastic anemia; MAC, myeloablative conditioning; RIC, reduced-intensity conditioning; CR, complete remission; PR, partial remission; NR, non-remission; MNC, mononuclear cell count; MRD; minimal residual disease; CMV, cytomegalovirus; EBV, EB virus; aGVHD, acute graft-versus-host disease.

### Associations between BAR and prognosis

**Table 2** displays the results of multivariable Cox regression analyses assessing the association between BAR and both ACM and NRM. When analysed as a continuous variable, elevated BAR demonstrated significant associations with increased ACM (HR=5.92, 95% CI 1.66-9.16; p=0.006) and NRM (HR=5.26, 95% CI 1.48-8.71; p=0.010) after adjustment for multiple covariates: age; sex; stem cell source; transplant type; conditioning regimen; ABO match; MNC count; CD34+ cell count; granulocyte engraftment time; CMV viremia; EBV viremia; haematological parameters (white blood cells, haemoglobin, platelets); biochemical markers (total bilirubin, creatinine); and clinical complications (pulmonary infection, intestinal, febrile neutropenia, aGVHD grade).

**Table 2:** Association between BAR and ACM and NRM in patients with aGVHD following allo-HSCT.

Characteristic	Total, n (%)	Event, n (%)	Crude model		Model 1		Model 2		Model 3	
			HR (95%CI)	*P*-value	HR (95%CI)	*P*-value	HR (95%CI)	*P*-value	HR (95%CI)	*P*-value
ACM										
BAR	109	69 (63.3)	7.29 (2.71~9.57)	<0.001	7.35 (2.64~9.44)	<0.001	7.65 (2.4~9.36)	0.001	5.92 (1.66~9.16)	0.006
BAR category										
T1	36	16 (44.4)	1(Ref)		1(Ref)		1(Ref)		1(Ref)	
T2	36	25 (69.4)	1.68 (0.89~3.16)	0.112	1.66 (0.88~3.16)	0.119	1.35 (0.65~2.81)	0.420	1.40 (0.61~3.22)	0.432
T3	37	28 (75.7)	2.61 (1.39~4.9)	0.003	2.63 (1.37~5.06)	0.004	2.33 (1.15~4.72)	0.019	2.19 (1.05~4.57)	0.037
Trend test				0.003		0.003		0.001		0.029
NRM										
BAR	109	72 (66.1)	6.91 (2.57~8.57)	<0.001	7.12 (2.56~19.82)	<0.001	7.14 (2.24~.79)	0.001	5.26 (1.48~8.71)	0.010
BAR category										
T1	36	18 (50)	1(Ref)		1(Ref)		1(Ref)		1(Ref)	
T2	36	26 (72.2)	1.57 (0.86~2.89)	0.145	1.57 (0.85~2.9)	0.150	1.31 (0.65~2.67)	0.449	1.40 (0.62~3.14)	0.417
T3	37	28 (75.7)	2.42 (1.31~4.45)	0.005	2.47 (1.32~4.65)	0.005	2.22 (1.11~4.42)	0.023	2.07 (1.01~4.22)	0.046
Trend test				0.004		0.005		0.020		0.040

BAR, blood urea nitrogen to albumin ratio; T1, BAR (0.03-0.13); T2, BAR (0.13-0.27); T3, BAR(0.27-1.32);ACM, all-cause mortality; NRM, non-relapse mortality; aGVHD, acute graft-versus-host disease; allo-HSCT, allogeneic hematopoietic stem cell transplantation; HR, Hazard Ratio; CI, Confidence Interval; Ref, reference.

Model 1: Adjusted for Age and Sex.

Model 2: Adjusted for Model1 and Indication for HSCT, Stem cell sources, Type of transplantation, Conditioning regimen, Days from transplantation to diagnosis, ABO match, MNC count, CD34^+^ cells count.

Model 3: Adjusted for Model2 and Granulocyte implantation time, CMV viremia, EBV viremia, White blood cells, Hemoglobin and Platelets, Total bilirubin, Creatinine, Pulmonary infection, Intestinal infection, Febrile neutropenia, aGVHD grade.

**Figure 2:**
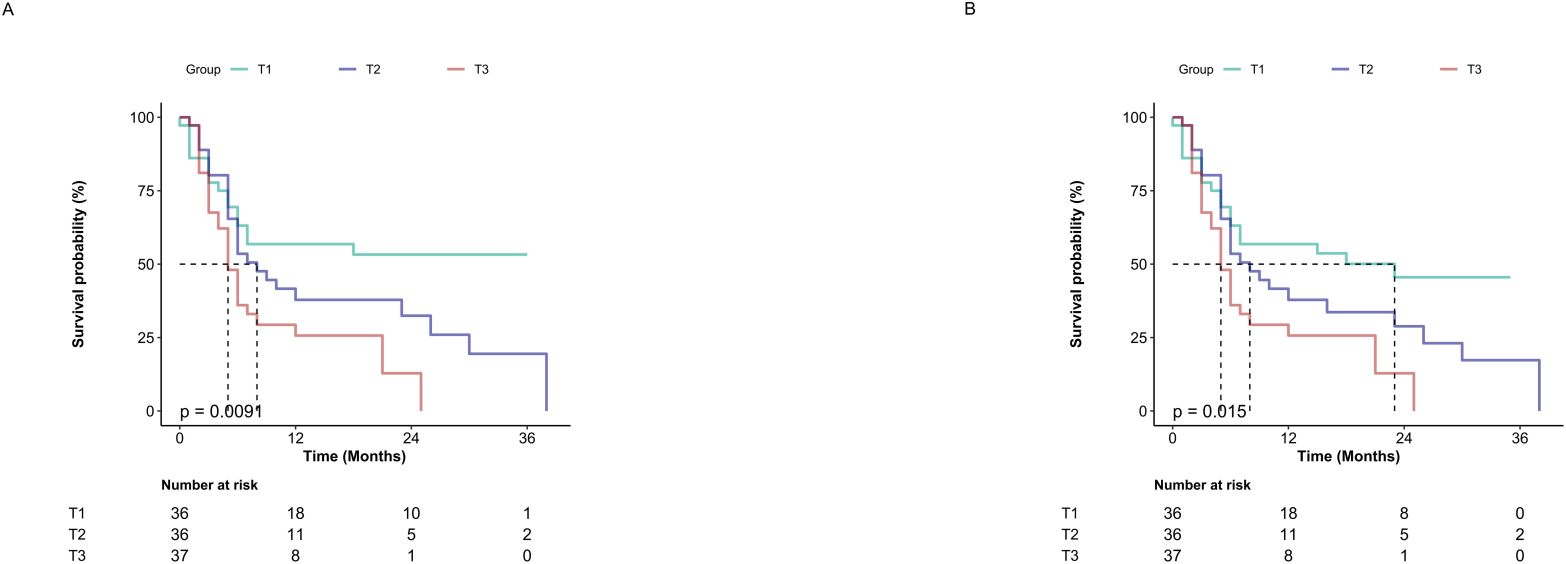
Kaplan-Meier survival curves for overall survival (OS) **(A)** and relapse-free survival (RFS) **(B)** in patients with aGVHD after allogeneic HSCT, stratified by BAR levels.

When BAR was evaluated as a categorical variable, the adjusted hazard ratios (HR) for BAR and ACM in T2(0.13-0.27) and T3(0.27-1.32) were 1.40 (95% CI: 0.61-3.22, *p*=0.432) and 2.19 (95% CI: 1.05-4.57, *p*=0.037), respectively, compared to individuals with lower T1(0.03-0.13). For NRM, the adjusted HR values for BAR in T2(0.13-0.27) and T3(0.27-1.32) were 1,40 (95% CI: 0.62-3.14, *p*=0.417) and 2.07 (95% CI: 1.01-4.22, *p*=0.046), respectively, when compared to those with lower T1(0.03-0.13). Significant positive trends were observed for both ACM (p-trend 0.029) and NRM (p-trend 0.040), indicating a dose-dependent relationship. This finding indicates a significant association between progressive BAR elevation and mortality outcomes. Supporting this observation, Kaplan-Meier curves confirmed inferior survival outcomes in the elevated BAR cohort, with significantly reduced overall survival (OS) (*p*=0.0091) and relapse-free survival (RFS) (*p*=0.015) (**Figure 2**).

### Subgroup and interaction analyses

**Figure 3:**
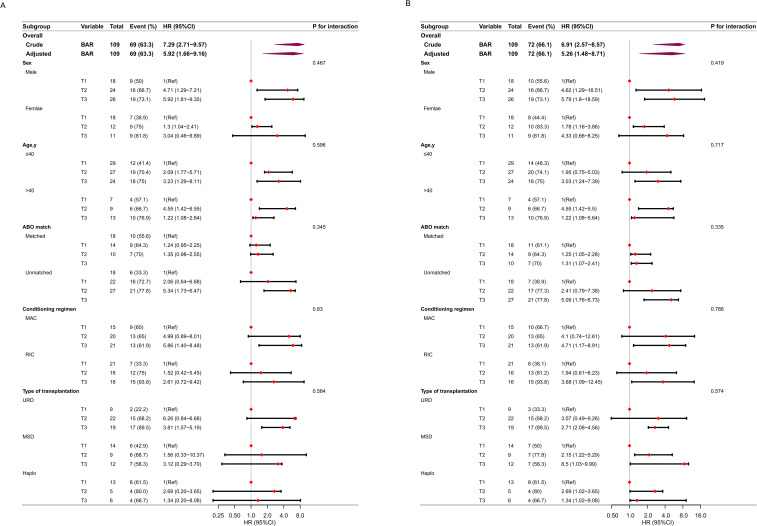
Association of BAR with ACM **(A)** and NRM **(B)** after multivariable adjustment, adjusted for: Age, Sex, Indication for HSCT, Stem cell source, Type of transplantation, Conditioning regimen, ABO match, Days from transplantation to diagnosis, MNC count, CD34+ cell count, Granulocyte infusion time, CMV viremia, EBV viremia, White blood cell count, Hemoglobin, and Platelet count,Total bilirubin, Creatinine, Pulmonary infection, Intestinal infection, Febrile neutropenia and aGVHD grade.

Subgroup and interaction analyses were conducted to assess the stability of the association between BAR levels and ACM and NRM (**Figure 3**), after adjusting for all covariates included in Model 3. Subgroup analyses based on age, sex, ABO match, conditioning regimen and type of transplantation revealed no statistically significant interactions (all interaction *p*-values > 0.05). The results from both the subgroup and interaction analyses confirmed the stability of the association between BAR levels and ACM and NRM.

Additionally, we performed subgroup and interaction analyses concerning MNC count; CD34+ cell count, EBV and CMV viremia, all of which yielded robust results. Due to space constraints in the main manuscript, these subgroup analyses are presented in **Supplementary Tables 1** and **2**.

### Sensitivity analyses

Sensitivity analyses excluding patients with survival duration <3 months confirmed the robustness of BAR-mortality associations (**Supplementary Table 3**). In the fully adjusted model (Model 3), elevated BAR as a continuous variable maintained significant associations with increased ACM (HR=5.77; 95%CI 1.96-6.64; *p*=0.004) and NRM (HR=5.96; 95%CI 2.49-9.69; *p*=0.005). Categorical analysis revealed a pronounced gradient effect across BAR tertiles. Compared to the reference tertile (T1: 0.03-0.13), the intermediate tertile (T2: 0.13-0.27) showed trends toward increased risk (ACM: HR=2.70, 95%CI 0.86-8.46, *p*=0.089; NRM: HR=2.49, 95%CI 0.86-7.20, *p*=0.093), the highest tertile (T3: 0.27-1.32) demonstrated substantially elevated risks for both ACM (HR=4.81; 95%CI 1.83-13.37; *p*=0.03) and NRM (HR=4.27; 95%CI 1.63-11.21; *p*=0.003).

## Discussion

This retrospective cohort study investigated the association between BAR and mortality in patients developing aGVHD after allo-HSCT. Our findings demonstrate that elevated BAR levels at aGVHD onset were significantly associated with an increased risk of mortality. These results suggest the BAR holds promise as a readily available prognostic biomarker, potentially enabling early identification of high-risk aGVHD patients and facilitating timely, targeted interventions to improve outcomes.

Allogeneic hematopoietic stem cell transplantation (Allo-HSCT) remains a potentially curative therapy for various hematologic malignancies and non-malignant disorders (20). Despite significant advancements in supportive care, conditioning regimens, and donor selection, post-transplant morbidity and mortality remain substantial concerns (23, 24). All-cause mortality (ACM) following HCT is influenced by a complex interplay of factors including relapse of the underlying disease, GVHD, infections, and organ toxicities (25, 26). Non-relapse mortality (NRM), specifically, represents a critical endpoint reflecting transplant-related complications and serves as a key metric for evaluating transplant safety and identifying modifiable risk factors (27). Accurately stratifying patients for their risk of NRM and ACM is paramount for tailoring preventative strategies and optimizing post-transplant care. Although BAR may reflect infection-driven systemic inflammation, its persistent association with ACM/NRM after adjusting for established infection biomarkers (e.g., procalcitonin) and confirmed infections (HR 1.24, p=0.004) suggests BAR captures pathology beyond mere infection severity. This may involve GVHD-specific mechanisms such as endothelial dysfunction (5), supporting BAR’s role as a prognostic marker for GVHD.

Current risk stratification models for post-HCT outcomes predominantly incorporate established factors such as age, performance status, comorbidity indices (e.g., Hematopoietic Cell Transplantation Comorbidity Index - HCT-CI), disease risk, donor type, and GVHD prophylaxis (28–37). While these models provide valuable prognostic information, they may not fully capture dynamic physiological changes occurring peri-transplant that could influence outcomes (28, 38). Nutritional and metabolic status, often assessed through serum albumin, has been recognized as a significant prognostic factor in HCT, with hypoalbuminemia associated with increased infection risk and poorer survival (39). Similarly, renal function, frequently monitored via blood urea nitrogen, creatinine, or estimated glomerular filtration rate (eGFR), is crucial, as renal impairment is a known contributor to NRM (19, 35, 36). The BAR, a simple metric calculated from routine laboratory tests, has emerged as a potential indicator of physiological stress in other critical care and medical contexts, reflecting a combination of catabolic state, renal dysfunction, and nutritional depletion (13, 14, 34). Preliminary studies in non-transplant settings suggest its prognostic value for mortality in conditions like sepsis, heart failure, and cirrhosis (13, 14, 37). Current aGVHD biomarker research prioritizes inflammatory mediators with conflicting roles (41), while our study establishes BAR as a novel metabolic-nutritional prognostic indicator, overcoming symptom-based and specialized testing limitations for real-world risk stratification in resource-limited settings.

Despite the established importance of both nutritional status (represented by ALB) and renal function (reflected partly by BUN) in HSCT outcomes, current prognostic models often treat these parameters in isolation (23, 24, 40). Existing research on biomarkers in HCT has focused on more complex or expensive assays (e.g., cytokines, genetic markers) (38, 42–47), while the prognostic utility of readily available, inexpensive, and composite metrics like BAR remains largely unaddressed. Furthermore, the optimal timing for assessing such a ratio relative to transplant (e.g., pre-transplant, at engraftment, post-engraftment during early recovery) requires investigation, as the peri-transplant period involves significant physiological fluctuations (38, 46–49). BAR serves as an integrative marker of metabolic burden and nutritional reserve insufficiency, identifying patients at heightened vulnerability to transplant-related complications beyond traditional risk assessments. This readily accessible tool enables early risk stratification, guiding intensified monitoring (e.g., renal/nutritional support) and targeted interventions (e.g., infection prophylaxis) to reduce mortality in high-risk individuals with elevated BAR. Beyond GVHD, BAR captures systemic burden across transplant settings, as evidenced by improved organ function correlating with survival post-autologous HCT for scleroderma, underscoring its trans-disease utility (50).

This study’s retrospective design and limited sample size constrain its generalizability. However, rigorous statistical methods and clinically significant hazard ratios support BAR’s prognostic relevance, warranting future prospective validation in larger cohorts. This retrospective study is susceptible to selection bias, unmeasured confounding, and heterogeneity in transplant protocols (e.g., donor source, conditioning regimens). BAR measurement timing variations relative to aGVHD diagnosis may affect precision, while unavailable infection severity scores and GVHD treatment details limit covariate adjustment. These constraints necessitate prospective validation through registered protocols (e.g., ClinicalTrials.gov).

This marker warrants validation in prospective, multicenter cohorts to confirm its prognostic generalizability. Further studies should integrate BAR with established biomarkers and multi-omics data through AI-driven models to enhance aGVHD mortality prediction accuracy. Ultimately, elucidating the molecular mechanisms linking BAR to aGVHD pathogenesis could uncover novel therapeutic targets for precision interventions.

## Conclusion

Based on our cohort of patients with aGVHD following allo-HSCT, this study demonstrates that elevated BAR levels independently predict increased mortality in allo-HSCT recipients with aGVHD. These findings support the potential utility of the BAR as a novel prognostic indicator in this setting. Consequently, this biomarker should inform the development and refinement of clinical management guidelines. Further prospective multicenter studies are imperative to provide robust validation of these results.

## Data Availability

The raw data supporting the conclusions of this article will be made available by the authors, without undue reservation.
