# Supplementary material for: Blood urea nitrogen-to-albumin ratio predicts mortality in acute graft-versus- host disease after allogeneic stem cell transplantation
Source: Front Immunol. 2026 Jul 16;17:1796065. doi: 10.3389/fimmu.2026.1796065 (PMC13421441; doi:10.3389/fimmu.2026.1796065)
Supplement: Supplementary file 1 [file Table1.docx]

**Supplementary Table 1**  Subgroup analyses of the association between BAR and ACM in aGVHD following allo-HSCT

| Subgroup | Variable | OS | | | *P*-Value | *P* for interaction |
| --- | --- | --- | --- | --- | --- | --- |
|  |  | Total | Event(%) | HR(95%CI) |  |  |
| MNC(10^8^/Kg） |  |  |  |  |  |  |
| ≤10 |  |  |  |  |  | 0.108 |
|  | T1 | 17 | 10 (58.8) | 1(Ref) |  |  |
|  | T2 | 11 | 8 (72.7) | 1.11 (0.01~2.12) | 0.062 |  |
|  | T3 | 12 | 8 (66.7) | 1.27 (0.03~2.50) | 0.251 |  |
| ＞10 |  |  |  |  |  |  |
|  | T1 | 19 | 6 (31.6) | 1(Ref) |  |  |
|  | T2 | 25 | 17 (68) | 3.07 (0.88~10.72) | 0.079 |  |
|  | T3 | 25 | 20 (80) | 6.10 (1.84~20.21) | 0.003 |  |
| CD34+(10^6^/Kg） |  |  |  |  |  |  |
| ≤6 |  |  |  |  |  | 0.995 |
|  | T1 | 20 | 8 (40) | 1(Ref) |  |  |
|  | T2 | 17 | 11 (64.7) | 1.16 (0.32~4.22) | 0.820 |  |
|  | T3 | 17 | 13 (76.5) | 2.50 (0.68~9.12) | 0.166 |  |
| ＞6 |  |  |  |  |  |  |
|  | T1 | 16 | 8 (50) | 1(Ref) |  |  |
|  | T2 | 19 | 14 (73.7) | 4.06 (0.77~21.45) | 0.099 |  |
|  | T3 | 20 | 15 (75) | 8.50 (1.43~50.65) | 0.019 |  |
| CMV viremia |  |  |  |  |  |  |
| No |  |  |  |  |  | 0.187 |
|  | T1 | 31 | 14 (45.2) | 1(Ref) |  |  |
|  | T2 | 26 | 16 (61.5) | 0.63 (0.23~1.76) | 0.378 |  |
|  | T3 | 28 | 20 (71.4) | 1.41 (0.51~3.91) | 0.508 |  |
| Yes |  |  |  |  |  |  |
|  | T1 | 5 | 2 (40) | 1(Ref) |  |  |
|  | T2 | 10 | 9 (90) | 7.04 (4.13~15.59) | <0.001 |  |
|  | T3 | 9 | 8 (88.9) | 7.54 (2.38~23.84) | 0.001 |  |
| EBV viremia |  |  |  |  |  |  |
| No |  |  |  |  |  | 0.220 |
|  | T1 | 30 | 14 (46.7) | 1(Ref) |  |  |
|  | T2 | 32 | 21 (65.6) | 1.77 (0.30~1.98) | 0.585 |  |
|  | T3 | 29 | 22 (75.9) | 1.84 (0.83~4.07) | 0.135 |  |
| Yes |  |  |  |  |  |  |
|  | T1 | 6 | 2 (33.3) | 1(Ref) |  |  |
|  | T2 | 4 | 4 (100) | 7.04 (14.13~15.59) | <0.001 |  |
|  | T3 | 8 | 6 (75) | 7.54 (2.38~23.84) | <0.001 |  |

BAR, blood urea nitrogen to albumin ratio; ACM, all-cause mortality; MNC, mononuclear cells; CMV, cytomegalovirus; EBV, Epstein-Barr Virus; T1, BAR (0.03-0.13); T2, BAR (0.13-0.27); T3, BAR(0.27-1.32); aGVHD, acute graft-versus-host disease; allo-HSCT, allogeneic hematopoietic stem cell transplantation.
