# Supplementary material for: Blood urea nitrogen-to-albumin ratio predicts mortality in acute graft-versus- host disease after allogeneic stem cell transplantation
Source: Front Immunol. 2026 Jul 16;17:1796065. doi: 10.3389/fimmu.2026.1796065 (PMC13421441; doi:10.3389/fimmu.2026.1796065)
Supplement: Supplementary file 2 [file Table2.docx]

**Supplementary Table 2**  Subgroup analyses of the association between BAR and NRM in aGVHD following allo-HSCT

|  | Variable | NRM | | | *P*-Value | *P* for interaction |
| --- | --- | --- | --- | --- | --- | --- |
|  |  | Total | Event(%) | HR(95%CI) |  |  |
| MNC(10^8^/Kg） |  |  |  |  |  | 0.136 |
| ＜10 | T1 | 17 | 10 (58.8) | 1(Ref) |  |  |
|  | T2 | 11 | 8 (72.7) | 1.11 (0.01~1.12) | 0.062 |  |
|  | T3 | 12 | 8 (66.7) | 1.27 (0.03~2.50) | 0.251 |  |
|  |  |  |  |  |  |  |
| ＞10 | T1 | 19 | 8 (42.1) | 1(Ref) |  |  |
|  | T2 | 25 | 18 (72) | 2.18 (0.70~6.78) | 0.176 |  |
|  | T3 | 25 | 20 (80) | 4.58 (1.45~14.5) | 0.010 |  |
|  |  |  |  |  |  | 0.814 |
| CD34+(10^6^/Kg） |  |  |  |  |  |  |
| ＜6 | T1 | 20 | 8 (40) | 1(Ref) |  |  |
|  | T2 | 17 | 11 (64.7) | 1.16 (0.32~4.22) | 0.820 |  |
|  | T3 | 17 | 13 (76.5) | 2.50 (0.68~9.12) | 0.166 |  |
|  |  |  |  |  |  |  |
| ＞6 | T1 | 16 | 10 (62.5) | 1(Ref) |  |  |
|  | T2 | 19 | 15 (78.9) | 2.69 (0.69~10.44) | 0.153 |  |
|  | T3 | 20 | 15 (75) | 5.54 (1.23~25.00) | 0.026 |  |
|  |  |  |  |  |  | 0.359 |
| CMV viremia |  |  |  |  |  |  |
| No | T1 | 31 | 15 (48.4) | 1(Ref) |  |  |
|  | T2 | 26 | 17 (65.4) | 0.81 (0.30~2.17) | 0.669 |  |
|  | T3 | 28 | 20 (71.4) | 1.46 (0.54~3.96) | 0.462 |  |
|  |  |  |  |  |  |  |
| Yes | T1 | 5 | 3 (60) | 1(Ref) |  |  |
|  | T2 | 10 | 9 (90) | 1.11 (0.56~5.98) | <0.001 |  |
|  | T3 | 9 | 8 (88.9) | 1.98 (1.04~7.61) | <0.001 |  |
|  |  |  |  |  |  | 0.111 |
| EBV viremia |  |  |  |  |  |  |
| No | T1 | 30 | 16 (53.3) | 1(Ref) |  |  |
|  | T2 | 32 | 22 (68.8) | 1.73 (0.30~1.79) | 0.491 |  |
|  | T3 | 29 | 22 (75.9) | 1.70 (0.78~3.68) | 0.179 |  |
|  |  |  |  |  |  |  |
| Yes | T1 | 6 | 2 (33.3) | 1(Ref) |  |  |
|  | T2 | 4 | 4 (100) | 1.34 (0.56~1.71) | <0.001 |  |
|  | T3 | 8 | 6 (75) | 1.56 (0.83~3.83) | <0.001 |  |

BAR, blood urea nitrogen to albumin ratio; NRM, non-relapse mortality; MNC, mononuclear cells; CMV, cytomegalovirus; EBV, Epstein-Barr Virus; T1, BAR (0.03-0.13); T2, BAR (0.13-0.27); T3, BAR(0.27-1.32); aGVHD, acute graft-versus-host disease; allo-HSCT, allogeneic hematopoietic stem cell transplantation.
