# Supplementary material for: Blood urea nitrogen-to-albumin ratio predicts mortality in acute graft-versus- host disease after allogeneic stem cell transplantation
Source: Front Immunol. 2026 Jul 16;17:1796065. doi: 10.3389/fimmu.2026.1796065 (PMC13421441; doi:10.3389/fimmu.2026.1796065)
Supplement: Supplementary file 3 [file Table3.docx]

**Supplementary Table 3** Association between BAR and mortality in patients with aGVHD

following allo-HSCT, excluding those with a survival time of less than 3 months

| Characteristic | Event,  n (%) | Crude model | |  | Model 1 | |  | Model 2 | |  | Model 3 | |
| --- | --- | --- | --- | --- | --- | --- | --- | --- | --- | --- | --- | --- |
|  |  | HR (95%CI) | *p*-Value |  | HR (95%CI) | *p*-Value |  | HR (95%CI) | *p*-Value |  | HR (95%CI) | *p*-Value |
| ACM |  |  |  |  |  |  |  |  |  |  |  |  |
| BAR | 42 (52.5) | 3.63 (1.89~9.12) | <0.001 |  | 5.36 (2.71~9.57) | <0.001 |  | 4.21 (2.06~9.99) | <0.001 |  | 5.77 (1.96~6.64) | 0.004 |
| BAR category |  |  |  |  |  |  |  |  |  |  |  |  |
| T1 | 8 (28.6) | 1(Ref) |  |  | 1(Ref) |  |  | 1(Ref) |  |  | 1(Ref) |  |
| T2 | 18 (66.7) | 2.58 (1.11~6.01) | 0.028 |  | 2.54 (1.08~5.96) | 0.032 |  | 1.99 (0.73~5.44) | 0.181 |  | 2.70 (0.86~8.46) | 0.089 |
| T3 | 16 (64) | 3.98 (1.66~9.55) | 0.002 |  | 4.90 (1.96~12.24) | 0.001 |  | 5.05 (1.8~14.2) | 0.002 |  | 4.81 (1.73~13.37) | 0.003 |
| Trend test |  |  | 0.002 |  |  | 0.001 |  |  | 0.002 |  |  | 0.003 |
| NRM |  |  |  |  |  |  |  |  |  |  |  |  |
| BAR | 45 (56.2) | 2.97 (1.76~4.68) | <0.001 |  | 3.93 (1.36~5.85) | <0.001 |  | 3.90 (2.69~7.54) | 0.001 |  | 5.96 (2.49~9.69) | 0.005 |
| BAR category |  |  |  |  |  |  |  |  |  |  |  |  |
| T1 | 10 (35.7) | 1(Ref) |  |  | 1(Ref) |  |  | 1(Ref) |  |  | 1(Ref) |  |
| T2 | 19 (70.4) | 2.21 (1.02~4.81) | 0.045 |  | 2.19 (1.00~4.81) | 0.05 |  | 1.70  (0.64~4.52) | 0.286 |  | 2.49 (0.86~7.20) | 0.093 |
| T3 | 16 (64) | 3.34 (1.48~7.55) | 0.004 |  | 4.15 (1.76~9.81) | 0.001 |  | 4.46 (1.68~11.83) | 0.003 |  | 4.27 (1.63~11.21) | 0.003 |
| Trend test |  |  | 0.003 |  |  | 0.001 |  |  | 0.003 |  |  | 0.003 |

BAR, blood urea nitrogen to albumin ratio; T1, BAR (0.03-0.13); T2, BAR (0.13-0.27); T3, BAR(0.27-1.32);ACM, all-cause mortality; NRM, non-relapse mortality; aGVHD, acute graft-versus-host disease; allo-HSCT, allogeneic hematopoietic stem cell transplantation; HR, Hazard Ratio; CI, Confidence Interval; Ref, reference.

Model 1: Adjusted for Age and Sex；

Model2: Adjusted for Model1 and Indication for HSCT, Stem cell sources, Type of transplantation, Conditioning regimen, Days from transplantation to diagnosis, ABO match, MNC count, CD34^+^ cells count；

Model3: Adjusted for Model2 and Granulocyte implantation time, CMV viremia, EBV viremia, White blood cells, Hemoglobin and Platelets, Total bilirubin, Creatinine, Pulmonary infection, Intestinal infection, Febrile neutropenia, aGVHD grade.
